# Factors contributing to intervention fidelity in a multi-site chronic disease self-management program

**DOI:** 10.1186/1748-5908-1-26

**Published:** 2006-10-26

**Authors:** Karen M Perrin, Somer Goad Burke, Danielle O'Connor, Gary Walby, Claire Shippey, Seraphine Pitt, Robert J McDermott, Melinda S Forthofer

**Affiliations:** 1 College of Public Health, University of South Florida, Tampa, Florida, USA; 2 Anthropology, University of South Florida, Tampa, Florida, USA; 3 College of Social Work, University of South Carolina, Columbia, South Carolina, USA

## Abstract

**Background and objectives:**

Disease self-management programs have been a popular approach to reducing morbidity and mortality from chronic disease. Replicating an evidence-based disease management program successfully requires practitioners to ensure fidelity to the original program design.

**Methods:**

The Florida Health Literacy Study (FHLS) was conducted to investigate the implementation impact of the Pfizer, Inc. Diabetes Mellitus and Hypertension Disease Self-Management Program based on health literacy principles in 14 community health centers in Florida. The intervention components discussed include health educator recruitment and training, patient recruitment, class sessions, utilization of program materials, translation of program manuals, patient retention and follow-up, and technical assistance.

**Results:**

This report describes challenges associated with achieving a balance between adaptation for cultural relevance and fidelity when implementing the health education program across clinic sites. This balance was necessary to achieve effectiveness of the disease self-management program. The FHLS program was implemented with a high degree of fidelity to the original design and used original program materials. Adaptations identified as advantageous to program participation are discussed, such as implementing alternate methods for recruiting patients and developing staff incentives for participation.

**Conclusion:**

Effective program implementation depends on the talent, skill and willing participation of clinic staff. Program adaptations that conserve staff time and resources and recognize their contribution can increase program effectiveness without jeopardizing its fidelity.

## Background

Self-management education programs can have a positive effect on patients' ability to control Type 2 diabetes and hypertension [1]. Given the current prevalence of these chronic diseases and associated health disparities, there is a need for community-based programs that improve health behaviors and health status. In attempts to expand such programs, health care providers often replicate successful self-management education interventions with populations that are different from those in the pilot study. In this context, program fidelity becomes an important consideration for replicating chronic disease management programs.

Experts argue that a degree of reinvention and adaptation is necessary to apply interventions in different settings and across diverse cultural groups [2]. Programs that display perfect fidelity to the original trial but are without a conceptual framework for adapting to different diseases, treatments, patients, providers, institutions, and cultures can be unrealistic in real-world clinical practice [2]. Nevertheless, when programs are not implemented according to protocol, researchers may conclude incorrectly that unexpected findings are due to theoretical or methodological problems with the intervention, rather than with its implementation [2,4]. Systematic monitoring of program delivery can contribute to the understanding of why interventions that may achieve high fidelity can succeed in some setting and fail in others.

Uncontrolled hypertension and Type 2 diabetes pose a major disease burden in the United States, including high morbidity, premature mortality, and enormous health care costs [5]. Much of this burden is preventable through management of blood pressure and blood glucose levels. However, successful disease management requires patients to make numerous behavioral changes, comply with complex therapeutic regimens, and undertake self-care activities [6,7].

Health literacy is the collection of skills that enable patients to function in the health care system, including basic reading ability and numerical skills [8]. Poor health literacy is more common among patients who have low educational attainment and among immigrants, older patients, and racial and ethnic minorities [8]. Low health literacy may contribute independently to the disproportionate burden of diabetes-related problems in disadvantaged populations [9]. A functional level of health literacy may be a necessary prerequisite for the systematic, effective interaction with providers required to complete chronic disease self-management [10].

The Florida Health Literacy Study (FHLS) program built upon lessons learned from several evidence-based programs to deliver a self-management intervention, which could be implemented with relative low cost by state Medicaid programs, that was tailored to the needs of low-income, culturally diverse populations with low rates of functional health literacy [11-14]. The intervention components included in the program were intended to represent low- to moderate-intensity variants of strategies previously shown to have sustained effectiveness. FHLS applied health literacy theories and techniques to widely accepted health education practices for implementation in community health centers. Program goals included improving participants' disease-specific knowledge, increasing self-care behaviors, and enhancing control of blood glucose and/or blood pressure levels.

### Purpose

The purpose of this FHLS report is to describe the challenges to achieving a balance between fidelity and implementation of a disease self-management program that was implemented in 14 community health centers in Florida. The intervention components discussed include: health educator recruitment and training, patient recruitment, class sessions, use of program materials, translation of program manuals, patient retention and follow-up, and technical assistance.

## Methods

### Community health centers

Following IRB approval, census data (2000) were used to identify potential federally qualified health centers. From the 48 health centers for which data were available, the 14 best matching pairs were identified based on comparable clinic services and programs, staffing configurations, and patient characteristics. Within each pair, community health centers were randomly assigned to implement the FHLS intervention or to continue with their "usual care" method of health education for Type 2 diabetes and hypertension.

### Community health center clinic staff and the university-based Project Staff (Project Staff)

Clinic staff consisted of currently employed nurses and primary care providers, as well as health educators who were hired specifically to implement the FHLS in their community health centers, which included teaching the program. The university-based project staff consisted of the Director of Implementation, the Project Coordinator, and several public health graduate students with supervision from the university faculty principal investigator.

### Community health center patients

According to the FHLS protocol, patients were recruited by physician referral. The physicians were given a triplicate copy, pre-printed prescription pad for the program. One copy stayed in the patient's medical chart; the second copy was put in the health educator's basket at the nurses' desk; and the third copy was given to the patient as a reminder to check-in with the health educator to schedule an appointment prior to leaving the clinic. If the patient did not make an appointment, the health educator retrieved the referral from the mail basket and called the patient at home to introduce the program and to schedule the initial appointment. The inclusion criteria for participation included a diagnosis of uncontrolled Type 2 diabetes, hypertension, or both. See Table 1 for a summary of the FHLS components, description and adaptation.

**Table 1:** Summary of FHLS Components

**FHLS components**	**Description**	**Adaptation**
Health educator recruitment	Placed ads in local newspapers; set minimal qualifications as health education experience.	Employed well-connected individuals from the local community with less than minimal qualifications.
Patient recruitment	Patient recruitment led by physicians or self-referral from clinic recruitment posters.	Clipped patient eligibility referral sheets to medical charts to prompt physician recruitment efforts.
Patient incentives	Incentives included a tote bag, glucose monitors and strips, and/or blood pressure monitor.	Incentives remained the same throughout the implementation.
Class sessions	Set curriculum.	Natural variation of teaching style using suggested curriculum.
Program materials	Posters, brochures, workbooks, low-literacy format, flipcharts for diabetes and hypertension, incentives (magnets, calendars, tote bags, medication pill box, medication compliance worksheet, food sheets).	All program materials were used and appreciated by patients and clinic staff. A few health educators enhanced materials, even though it was discouraged by project staff. Translated materials according to various Spanish-speaking cultures.
Program manuals	Developed by project staff and used in all clinic sites for standardized training and implementation.	The clinic training schedule was adapted to the needs of each clinic site.
Patient retention and follow-up	6 month commitment from patients.	Scheduled patient's health education appointments in conjunction with medical appointments.
Technical assistance	Offered support to health educators via e-mail or telephone, as needed.	Need for daily support to health educators was much greater than anticipated by project staff.
Staff incentives	Not included in the original design.	Frequently requested to increase staff buy-in.

#### Data collection

When assessing the fidelity of program implementation, researchers used documentation such as:

##### Logs of technical assistance and field notes

While providing technical assistance, the project staff collected data from logs of support and technical assistance requests made by health educators and health center staff via e-mail or telephone, and field notes from site visits with the health educators at 2–3 week intervals.

##### Class observations

During site visits, project staff met community health center staff to discuss any problems and to observe a minimum of three classes taught by the health educator. Prior to conducting any class observations, a standardized checklist was developed. Following each class observation, the project staff shared observations with the health educators to encourage their strengths and identify strategies for improving teaching techniques in future classes.

##### Patient narratives

The patient narratives offered the project staff information on program implementation from the patients' perspectives (i.e., was the program received as intended). The narratives also offer insight into patients' opinions of program adaptation/reinvention.

##### Exit interviews with health educators

Since the health educators served as the front-line clinic staff, they were interviewed by the project staff regarding their role, level of satisfaction with the program, and suggestions for improving the program in the future.

### Data analysis

Qualitative data from technical assistance logs, field notes, class observations, and exit interviews along with narratives sent in by the health educators were entered into Ethnograph^® ^qualitative analysis software. The project staff reviewed the printed data files, developed a coding scheme and agreed upon common themes that emerged from the qualitative data.

## Results

### Health educator recruitment and training

The project staff trained the health educators in one-on-one or small group formats at the convenience of the community health center. The training was individualized to accommodate the wide range of knowledge and expertise related to health education in general – and to Type 2 diabetes and hypertension, in particular. Although the mode of training delivery varied by site, the clinic staff manual and checklist were used to promote consistency of content across each site [16].

Review of this program implementation across sites underscored the challenges associated with recruitment of health educators, particularly in rural counties, due to a lack of qualified applicants. Organizations planning for program replications are encouraged to allocate considerable time and energy to recruitment in order to maximize their ability to identify staff with bilingual skills, a proven record of accomplishment of organizational and interpersonal skills, and general knowledge of the health problems relevant to the program. Conducting health educator training in a small group setting is likely to be more timely and affordable than one-on-one training, although group-based training is not without its own array of scheduling challenges.

Since the organizational chart of the clinic sites varied greatly, compliance with the study protocol varied also. In some clinics, the top-tier management and supervisor of the health educator were located in a central office as far as 25 miles away. In other clinics, the health educator's immediate supervisor was housed in the same clinic. The university-based project staff noted several advantages and disadvantages of both models. For some health educators, the distance provided the health educators with the opportunity to work more independently and, thus, become part of the clinic staff, but for others the distance created daily e-mails and telephone calls from supervisors trying to manage the project from a distance. In some situations, the distance delayed the transfer of communication and the receipt of supplies from the central shipping depot. When the supervisor was on-site, the health educator had the opportunity to receive immediate, first-hand communication and feedback that enhanced the fidelity of the program implementation. Consistent on-site supervision of the health educators is recommended as advantageous to the overall implementation process.

Upon the completion of their training, the health educators trained the other community health center clinic staff about FHLS. To support a consistent implementation pattern at the onset, project staff was present to assist with possible questions. Though the clinic staff training was designed for a group setting, this format proved to be difficult given clinic schedules. Therefore, most clinic staff training was provided over the course of one day in a small group or one-on-one format. Due to the critical role of the clinic staff in the program, the health educators worked hard to streamline the training in order to maximize the limited staff time available, while allowing time for questions and thus achieving buy-in from the staff.

Training of clinical staff represents an area of this program implementation in which adaptation and reinvention should be recognized as key to program success. Each organization has a unique staff culture with respect to the acceptance of new initiatives, and structuring training of clinical staff in a way that acknowledges and stimulates interest and commitment to the program's objectives.

### Patient recruitment

The program model was designed for patient recruitment led by physician referrals, however, it was not always practical to get full participation from physicians who work in busy community health care centers. Therefore, midway through the study the health educators were encouraged to adapt recruitment strategies for their setting in a variety of ways so as to achieve the maximum number of enrolled patients. The FHLS program included a recruitment poster to be posted in the clinics, encouraging patients to self-refer to the program. In addition, the health educators clipped a pre-screening eligibility referral sheet to medical charts as a prompt for the provider, rather than using the pre-printed prescription pad. Both changes increased the number of patients recruited.

### Class sessions

The project staff established a set of criteria prior to observing the health educators teach their classes, including issues such as organization of classroom setting, knowledge of curriculum, cultural and low-literacy competency in teaching, ability to respond to student questions, content and process.

Systematic observation of the class sessions underscored the extent of natural variation in the style of delivering the same content across sites. For example, some health educators were much more animated in their teaching style, while others were more serious in their approach to the content. Overall, the observers noted a high attendance rate among the patients that confirms patient appreciation of the new knowledge and understanding about management of their chronic disease, which was irrelevant of the teaching style of the health educator.

### Program materials

Several health educators mentioned the need to supplement the curriculum with a lesson on how to read food labels, while others mentioned the need for a recipe card file box. One health educator thought that a monthly newsletter would serve as a booster lesson and encourage patients to maintain diet and lifestyle changes that were initiated while attending the health education modules.

Since community health centers operate on limited budgets, there is rarely enough money to produce 4-color, matching health education materials in a low-literacy format that were provided for the FHLS. The nurses liked having the tri-fold brochures that explained Type 2 diabetes and/or hypertension in either English or Spanish. Also, there was a strong positive response from the health educators for the program materials, including flipcharts for diabetes and hypertension used to teach the classes.

### Program manuals

Translation from one language to another is the most obvious form of program adaptation [15]. However, there are numerous challenges when a health education program is translated. The health educators and patients represented various Spanish-speaking cultures, including Mexican-American, Cuban, and Central American. The formal Spanish translation of the program materials did not always match the dialect of a particular country of origin.

The main concern related to the food/diet section. The food list was translated into Spanish, but because there are many different Spanish-speaking cultures in Florida, some of these foods may have unique idiomatic names. Also, the list of foods did not focus on foods common to specific Spanish-speaking cultures, such as tortillas, plantains, rice and beans.

### Patient retention and follow-up

The program required a six-month commitment from the participating patients, and health educators experienced difficulty in tracking patients for this long. In addition, several of the community health centers serve a migrant population, which makes the retention and follow-up even more challenging for the health educators:

"I have a study patient who is a migrant worker. When he missed class today, I called and was told that he was relocated to another migrant camp. He left no follow-up address."

The highest levels of patient retention and compliance with follow-up appointments were noted in the clinics that scheduled the patient's health education appointments in conjunction with their medical appointments. Such a system reduced barriers related to lack of transportation, being absent from work, and confusion with multiple appointments.

### Technical assistance

Project staff offered support to the health educators via e-mail or telephone conversations on a daily basis, as necessary. The health educators expressed appreciation for the technical support and frequent personal visits, which allowed project staff to track the progress of the enrollment numbers while gathering valuable information on the actual implementation process.

## Discussion

Use of consistent implementation procedures and program materials promotes the fidelity of the program intervention. However, several important program adaptations were identified to ensure optimal participation in a multi-cultural, low-income clinic population. These adaptations included:

▪ Alternate methods of patient recruitment. In addition to physician recruitment, the development of physician-prompt systems and posters for self-referral increased client participation in the program. In considering program replication, researchers should weigh the benefits of strict fidelity to program protocols versus the benefits of reaching the maximum number of clients through enhanced referral methods.

▪ Customization of clinic staff training delivery format. The project staff realized that clinic staff training and participation were crucial to the implementation success. To maximize clinic staff knowledge and support of program goals, project staff developed a variety of training methods and processes. It should be stressed that future program implementations should allow sufficient planning and scheduling time to enable outreach to all clinic staff with a minimum number of scheduled trainings.

▪ Patient and staff incentives. Both patients and clinic staff appreciated incentives for program participation. In the case of clients, patient incentives were part of the original program design; however, clinic staff incentives were not. Developing clinic staff incentives enhanced compliance across the multi-site program implementation and included catered meals and tote bags.

▪ Culturally appropriate terms. Use of clinic staff to verbally translate Spanish-language materials into culturally and linguistically appropriate terms for their specific patient population.

▪ Recognition of unique institutional settings. Project staff determined that on-site supervision would provide the greatest degree of program effectiveness. However, this supervisory model was inconsistent with the organization of several participating clinics. Therefore, the project staff used a variety of supervisory models, depending on the existing organizational structure of the clinic. This model enabled successful program implementation without disrupting the day-to-day operations of participating clinics.

High levels of program implementation fidelity depend on the type of program, the mode of delivery, and the interaction between them [16]. Health literacy programs delivered in a multicultural, public clinic context must reflect the reality of that environment. To be efficacious in clinical settings, implementation programs must take into account the diversity of patients, providers, institutions, and cultures involved in the intervention [3]. Ultimately, effective program implementation depends on the talent, skill and willing participation of clinic staff. Program adaptations that conserve staff time and resources and recognize their contributions can increase program effectiveness without jeopardizing its fidelity.
